# Personal perspectives: Infant pain—A multidisciplinary journey

**DOI:** 10.1002/pne2.12017

**Published:** 2020-04-28

**Authors:** Ruth Eckstein Grunau

**Affiliations:** ^1^ Department of Pediatrics Faculty of Medicine University of British Columbia Vancouver BC Canada; ^2^ Brain, Behaviour and Development BC Children’s Hospital Research Institute Vancouver BC Canada

**Keywords:** brain, development, infant, pain, preterm, stress

## Abstract

Understanding of infant pain has been transformed in the past 30 years. From assumptions that newborns were insensitive to pain, fundamental work established not only the infants perceive pain, but also there are critical windows in which pain can have long‐lasting consequences. My multidisciplinary work revealed that repetitive pain exposure during the late 2nd and 3rd trimesters of fetal life “ex‐utero” in infants born very preterm is related to long‐term adverse associations with altered brain development, programming of stress systems, and thereby neurodevelopment. Here, influences will be described, discovery research summarized, and evidence of biological pathways proposed.

## 1 HISTORY

### 1.1 Key scientific events

Given the dearth of knowledge of infant pain at that time, it was exhilarating to undertake research in this field in the 1980s. The multidisciplinary nature of this field was evident from the beginning; my background in developmental psychology was ideal, as complementary to other researchers in the field. Maria Fitzgerald, a developmental biologist, was in the forefront of discovering and elucidating the neurobiology of early pain in immature rodents and human neonates,^1^, ^2^ and continues to lead this field. Anand (pediatric critical care medicine) published the landmark study showing neonates benefit from anesthesia during surgery,^3^ and Celeste Johnston^4^ (nursing) began to address infant pain measurement. Ken Craig—a clinical psychologist—was already a well‐established expert in facial action coding in adult pain. As his PhD student, together we applied facial coding principles to newborns, developing the first quantitative reliable validated measure of behavioral reactivity to pain in infants.^5^, ^6^ At that time, the Neonatal Facial Coding System (NFCS) was viewed as the gold standard for infant pain, comprising objective‐specific discrete facial movements and sleep‐wake state changes. The NFCS substantively influenced the field of infant pain assessment, as the NFCS core facial actions and sleep/wake state coding became incorporated into many of the multifactorial scales which followed, for example the Premature Infant Pain Profile.^7^

### 1.2 Personal timeline

Modern high technology intensive care for extremely preterm infants was established in the early 1980s. I completed my PhD in 1985, and became the first psychologist in the newly founded Neonatal Follow‐up Program at BC Children's Hospital in Vancouver. Our Research Institute was incorporated in 1995 in conjunction with the University of British Columbia (UBC) and the BC Children's and Women's Hospitals. I was invited to join as a clinical researcher—awarded one day a week “protected time” for research in 1996‐1998. As of 1999, I was very fortunate to receive a series of highly competitive salary awards continuing to this day. In 2001, I moved from the clinical to the academic track in the Division of Neonatology, Department of Pediatrics UBC, with a primary focus on research, becoming a full professor in 2006. I am indebted to directors of our Research Institute, especially Aubrey Tingle, Stuart McLeod and currently Wyeth Wasserman for their support. I am grateful to the neonatologists in my division, especially Mike Whitfield and Anne Synnes, and to my wonderful trainees at every level, who contribute so much to my research program. It has been an honor and privilege to have the opportunity to mentor so many fantastic young emerging scientists. In this overview, I can only highlight key points in my research, thus work of many outstanding trainees will be understated or not mentioned due to space. Similarly, I emphasize that my research program is multidisciplinary, which greatly enriches all aspects, but can only be touched on here.

Medical advances led to greatly increased survival of tiny newborns starting in the 1980s. Concurrently, extensive research worldwide described poorer cognitive, motor, and behavioral development in very preterm children.^e.g.^ ^8^ However, at that time, beyond a few medical risk factors such as lower gestational age, severe brain injury, and persistent lung disease, the etiology of altered neurodevelopment was large unclear. At the same time, evidence for pain perception in newborns was becoming established. One of my research directions was to characterize autonomic, stress hormone, and behavioral reactivity to acute procedural pain in infants born very preterm and full‐term,^9^ and in infancy after hospital discharge.^10^, ^11^ With my first post‐doc Sara Morison, we discovered how disparate behavioral and physiological responses are in both full‐term and preterm neonates.^12^ With my PhD student Liisa Holsti, we found that, surprisingly, pain reactivity in very preterm neonates was not a function of the extent of tissue damage, but rather was affected by previous events with in the past hour, past day, and cumulatively during the NICU stay.^e.g.^ ^13^ This was consistent with Maria Fitzgerald's work that established the importance of sensitization in pain reactivity of immature neonates in both rats and humans.^14^ Alongside those studies, I showed that pain sensitivity as reported by parents differed in children born extremely preterm compared with healthy full‐term.^15^ Subsequently, we established that indeed neonatal pain (quantified as the number of invasive procedures in the NICU), beyond clinical factors of prematurity, was associated with later pain sensitivity,^16^ neurodevelopment, and behavior during infancy and childhood.^17^, ^18^ Many clinical factors such as gestational age at birth, illness, duration of respiratory support on mechanical ventilation, infections, and surgeries are correlated with how many invasive procedures are conducted on infants undergoing intensive care. Therefore, we controlled statistically for these factors in our cohort studies as far as possible.

My next step was to look at the etiology of altered neurodevelopment in children born very preterm, and underlying pathways. Repetitive pain exposure (~10 invasive procedures daily)^19^ is developmentally unexpected in very preterm neonates during fetal life outside the protective intrauterine environment, during a period of the most rapid brain development and programming of stress systems. Several streams of my earlier experience came together to fundamentally influence the next directions of my research. Before my PhD, I worked on research on brain activity during cognition in children born preterm using task‐related electrophysiology (EEG), under Morton Low (neurologist and neurophysiologist) in the Faculty of Medicine at UBC.^20^, ^21^ In the Neonatal Follow‐up Program at BC Children's Hospital, conducting neurodevelopmental assessments on children born extremely preterm, I was struck by their high anxiety displayed during cognitive challenges, even when they had normal intelligence. At that time, a major body of basic animal literature was revealing long‐term effects of fetal and early‐life *stress,* leading to high anxiety behaviors, and poorer learning and memory in adulthood. These long‐lasting changes caused by various stressors in rodent models were shown to be mediated by the hypothalamic‐pituitary‐adrenal (HPA) axis and brain (primarily the limbic system) (eg, see Matthews for a review^22^) This body of animal studies greatly influenced my thinking about potential pathways for how repetitive procedural pain and stress in the NICU might contribute to the long‐term poorer cognition, behavior, and motor function evident in children born very preterm, beyond clinical factors related to prematurity. To address complex mechanisms, over the next few years, I sought expert multidisciplinary collaborators, beginning with HPA axis (neuroscientist Joanne Weinberg) and brain (neurophysiologists Hal Weinberg and Urs Ribary, radiologist Ken Poskitt), and then Steven Miller (neurologist).

In the early 2000s, magnetoencephalography (MEG) neuroimaging was becoming established. Hal Weinberg at Simon Fraser University (SFU) was in the forefront of MEG, and had the first one in Canada. Stuart McLeod included me with a small group of researchers invited to attend a meeting on MEG at SFU. Thanks to my EEG background, I immediately appreciated the huge potential for MEG to elucidate brain function. I formed a collaboration with this SFU group, including Urs Ribary who contributed greatly to co‐supervising Sam Doesburg, my new postdoctoral fellow who had acquired expertise in MEG during his PhD research. Further, I am indebted to Ken Poskitt who made it possible for me to acquire research MRIs on this same cohort of children born very preterm at age 7‐8 years, long before we had a MRI research scanner at our site. MRI remains the “gold standard” for brain localization and MEG for fine‐grained timing of oscillatory brain activity, thus having both on the same children was invaluable. Later, Steven Miller (neurologist and international leader in advanced quantitative neonatal brain imaging) introduced new methods at our site to image sick small infants using a MRI‐compatible incubator to study brain development across the period of pain/stress exposure in the NICU using serial MRI (early in life and again at term equivalent age). We were the first site in Canada to acquire this methodology, and even today only a few centers worldwide have this capacity to conduct neuroimaging early in preterm life. Miller and I have since enjoyed a long‐standing highly productive collaboration.

To further address pathways underlying long‐term effects of pain and individual variation in impact among infants born very preterm, I approached experts in genetics who specialized in various systems, including stress and epigenetics (Angela Devlin), as well as inflammation (Stuart Turvey). Examining possible effects of morphine exposure for pharmacologic pain management on neurodevelopment and behavior has been woven into my cohort studies over the years. Most recently, together with experts Bruce Carleton and Colin Ross, we have begun to address pharmacogenomics of morphine metabolism in relation to individual variation in child behavior.

All Grunau studies cited in this manuscript were approved by the Clinical Research Ethics Boards at the University of British Columbia and the BC Children's & Women's Hospitals.

## 2 PATHWAYS OF LONG‐TERM “EFFECTS” OF PAIN‐RELATED STRESS IN VERY PRETERM NEONATES

**FIGURE 1:**
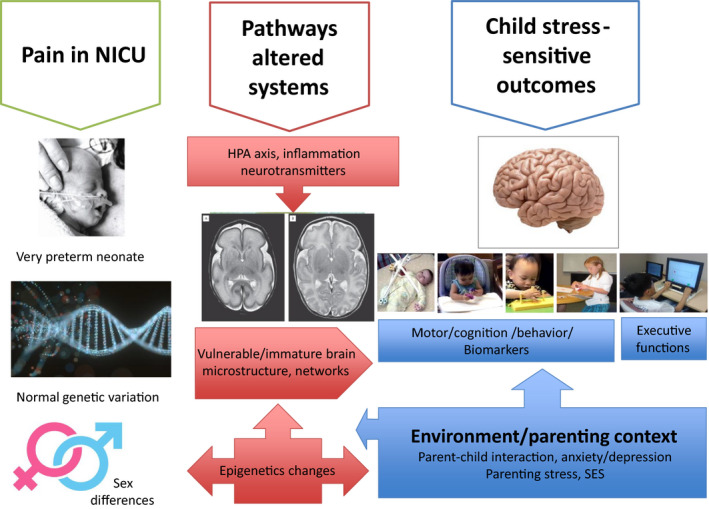
Schema of Grunau research program

Over the past 10‐15 years, we established that neonatal pain/stress in very preterm neonates is related to altered brain development and cortisol (stress hormone) regulation, and thereby affects neurodevelopment and behavior in this vulnerable population. Infants born very preterm are exposed to on average ~10 invasive procedures per day. Our human cohort studies converge with animal research in showing a developmentally vulnerable window of the immature developing brain and stress systems. My research utilizes clinical cohorts. With this approach, we are able to identify associations, always being mindful that this is not evidence of causality. Thus, the importance of identifying convergence between clinical cohort studies and the experimental animal literature to establish causal relationships cannot be overstated. My focus became to identify biologically plausible pathways underlying long‐term effects of neonatal pain on neurodevelopment of infants born 2‐4 months early (24‐32 weeks gestation). Several potential pathways including brain, stress hormone changes (indexed by cortisol) and interactions with the immune system (inflammation), and epigenetic changes appeared to be promising directions for effects of pain in very preterm neonates (see Figure 1). Targeted study of normal genetic variation was key to identifying risk and resilience to long‐term effects of neonatal pain.^23^, ^24^

### 2.1 Stress regulation

Cortisol is the primary stress hormone in humans. Cortisol levels have been studied extensively in the NICU, due to close inter‐relationships with illness and immune function. My work was the first to show that in very preterm infants, exposure to pain/stress (adjusted for clinical factors of prematurity) is related to altered cortisol levels from infancy to school age.^23^, ^25^, ^26^, ^27^, ^28^ Further, with post‐docs Haley and Tu, together with colleague Joanne Weinberg, we found the pattern of cortisol expression is linked to attention and behavior.^28^, ^29^ In the NICU, we found in our early studies of pain/stress reactivity that lower cortisol reactivity to stress at 32 weeks postmenstrual age was associated with higher number of invasive procedures since birth, after accounting for confounders such as early illness severity.^27^ There were numerous studies of cortisol during childhood in the general population by others, but a dearth of knowledge in children born very preterm. We compared cortisol levels longitudinally in a cohort born very preterm, with healthy full‐term controls as a comparison group also recruited at birth.^25^ Surprisingly, developmental shifts were seen across time, with higher baseline cortisol levels in the extremely infants at 8 and 18 months corrected age (CA) compared with full‐terms, followed by lower levels at school age.^23^ This is consistent with studies in other populations, which have established that chronic stress eventually can lead to down‐regulation of cortisol over time.^30^ Susanne Brummelte post‐doc, studied salivary cortisol expression during cognitive challenge and diurnal patterns. We found cortisol levels across cognitive challenge were related to greater anxiety behaviors at 18 months CA^31^ and at 7‐8 years.^32^ Higher exposure to neonatal pain was related to cortisol levels in infancy, toddlerhood and at school age. Sex differences became apparent at age 7‐8 years, but not earlier. Others have recently shown similar findings of lower cortisol levels in adolescents and adults born very preterm compared with full‐term,^33^ thus results of studies converge.

### 2.2 Brain

Many studies have established differences in brain structure and function between children, adolescents and adults born very preterm compared with full‐term. Our work focused on whether neonatal pain/stress contributes to altered brain development, above and beyond clinical factors related to prematurity, and how this might occur. In a series of neuroimaging studies in two independent cohorts in Vancouver, we established associations between extent of pain‐related stress exposure in the NICU and brain development. Above and beyond neonatal clinical factors related to prematurity, Brummelte and Zwicker as post‐docs, found neonatal pain/stress was related to white matter and grey matter^34^ and corticospinal tract maturation^35^ in the neonatal period. In a separate cohort, Doesburg and Ranger post‐docs and Vinall a PhD student showed neonatal pain/stress predicted white matter maturity,^36^ cortical thickness,^37^ regional cerebellar volumes,^38^ and neuronal activity^39^, ^40^, ^41^, ^42^ at school age, and in turn, cognitive function and behavior. Recently, Doesburg with his students has extended this work.^40^, ^42^ Importantly, these changes to brain structure and function were above and beyond relationships with known clinical risk factors related to prematurity.

The thalamus is a relay hub for sensory input from the periphery to the cortex; therefore, this brain region is of particular interest in understanding long‐term effects of early pain. At age 7‐8 years, we found that greater exposure to neonatal pain (especially in children born 24‐28 weeks GA) was associated with altered MEG oscillatory activity^39^, ^40^, ^41^, ^42^ which is generated in the thalamus and reflects functional thalamocortical connectivity. Recently, we established that structural thalamic growth on MRI from early life to term equivalent age is affected by greater exposure to neonatal pain, in two new independent cohorts in Vancouver and Switzerland that underwent serial MRI in the neonatal period.^43^, ^44^ Importantly, our findings in infancy and at school age converge to identify the importance of pain primarily in a vulnerable period of extreme prematurity (24‐28 weeks GA), structurally and functionally affecting thalamic and thalamocortical development.

Reasons for the particular vulnerability of the extremely preterm brain to pertubations induced by procedural pain/stress are complex and multifaceted. Invasive procedures are known to trigger a cascade of behavioral, physiological, hormonal, inflammatory, hemodynamic, and EEG responses.^45^ These changes potentially can initiate excitotoxic changes in the brain, affecting maturation of oligodentrocytes (that produce the myelin sheath affecting speed of transmission), as well as development of thalamocortical connections which are particularly vulnerable in the 24‐28 postmenstrual weeks developmental window when this process is underway initially.^45^ Our conclusions that pain/stress likely plays a role in brain dysmaturation, particularly in infants born extremely preterm (≤28 weeks gestational age) appear to be biologically plausible.

### 2.3 Genetics

Another way to examine potential pathways underlying differential development of humans exposed to early stress, gene by environment interactions has long been viewed as a useful approach. This allows for identification of potential variation in risk and resilience, as well as systems involved.

#### 2.3.1 Genetic variation in regulation of neurotransmitters interacts with early‐life pain/stress

Epigenetic mechanisms regulating gene expression may contribute to diversity in behavioral outcomes^46^ and in long‐term changes in HPA and brain function^47^ following early‐life stress. Our work established “proof of principle” that neonatal pain/stress interacts with targeted genetic variation in regulation of serotonin and dopamine,^24^, ^48^ and that epigenetic processes may contribute to mediating long‐term effects on outcomes.^24^

#### 2.3.2 Neonatal pain/stress and inflammatory processes

We are the first to show that genetic variants in regulation of inflammation modify the association of neonatal pain/stress and cortisol activity in very preterm boys (but not girls) at 7 years, controlling for concurrent stressors.^23^

Given the close relationship between the stress and immune systems, I sought a way to examine whether the association between neonatal pain exposure and changes in later cortisol regulation might be affected by inflammatory factors implicating immune function. I approached Stuart Turvey, an expert in inflammation in pediatric diseases. Together, we examined normal genetic variations in the NFKBIA promotor region, involved in regulation of inflammatory responses. We found that in very preterm children, the relationship between exposure to neonatal pain/stress (adjusted for clinical confounders) and hair cortisol levels at age 8 years was moderated by the NFKBIA genotype, but only in very preterm boys, not girls. Moreover, children with the minor allele were associated with higher secretion of inflammatory cytokines (IL6, TNFα) at age 7‐8 years.23 Sex differences in long‐term effects of early pain and stress are frequently found in rodents, and our work points to fruitful possibilities for future research in humans to understand pathways that may affect males and females differentially.

#### 2.3.3 Neonatal pain/stress and neuroplasticity

Brain‐derived neurotrophic factor (BDNF) regulates neuronal survival and plasticity and is brain‐protective. We discovered that in boys (but not girls), variation in the BDNF gene moderated the relationship between neonatal pain/stress and cortisol reactivity to executive function tasks at 7 years.^49^ Together with Angela Devlin, we targeted genes known to be involved in expression of neurotransmitters (serotonin transporter *SLC6A4*, dopamine (Catechol‐O‐methyltransferrase *COMT*) and brain‐derived neurotrophic factor (*BDNF*), all of which are important in the developing brain formation of neural networks underlying cognition and behavior. Moreover, COMT is related to pain sensitivity. Indeed, we found that normal variation in each of these genes moderated associations between extent of exposure to pain‐related stress in relation to stress regulation (hair cortisol) at school age, and in turn with behavior and cognition. We found sex‐specific effects in these studies, for example, the *BDNF* gene variant moderated the relationship between neonatal pain and *cumulative* stress (hair cortisol) only in very preterm boys; however, in boys and girls with the Met allele (lower BDNF availability), higher stress *reactivity* (salivary cortisol) was related to lower IQ.^49^ Our work revealed that in very preterm children without major brain injury or cognitive impairment, genes involved with regulation of neurotransmitters and the neurotrophin BDNF identified greater or lesser risk for adverse effects of early pain. This approach, first, contributed to revealing some potential ways that pain could “get under the skin” to affect systems, and second, started to suggest genetic factors in vulnerability to early pain that may contribute to the variability in outcomes related to preterm birth.

Recently, we examined a panel of genes related to morphine metabolism in relation to internalizing behavior, and found markers that might have the potential to identify infants highly sensitive to adverse effects of morphine.^50^ Major multisite studies are needed to address the role of genetics, including polygene interactions and differential sex effects.

### 2.4 Brain and genetics

We recently discovered that neonatal pain/stress is differentially related to regional brain volumes in the limbic system and thalamus, using MRI. We found that neonatal clinical factors and genotypes (*BDNF, SLC6A4, COMT*) were associated with volumes of hippocampal subregions, tracts, basal ganglia, thalamus, and amygdala.^48^ After controlling for clinical risk factors and total brain volume, greater exposure to neonatal invasive procedures was associated with lower volumes in the amygdala, thalamus, and hippocampal subregions. More surgeries, days of ventilation, and lower GA were also related to smaller volumes in various subcortical regions. These reduced volumes were in turn differentially related to poorer cognitive, visual motor and behavioral outcomes. This is the first work to our knowledge, to attempt to integrate genetic moderators with relationships between early pain and brain development.

### 2.5 Epigenetic changes

Epigenetics refers to alterations in gene expression that change the phenotype, for example through changes in DNA methylation. Many factors can trigger these changes, including early‐life stress and adversity, and chronic pain.^51^ My work with Angela Devlin was the first study (to our knowledge) to examine whether neonatal pain‐related stress exposure in preterm infants might be mediated by epigenetic changes (methylation) in the serotonin transporter. Serotonin is involved in emotion, mood, memory, and HPA axis function, as well as other systems. We found that very preterm children had significantly higher methylation in the SLC6A4 promotor compared with term‐born children at age 7 years, and that this higher methylation was associated with more behavior difficulties in the very preterm children.^24^ A group in Italy has refined and extended this work.^52^ Taken together, these findings suggest that methylation of the SLC6A4 gene may differ between preterm and term‐born infants may be affected by pain exposure among preterm infants during NICU care, and that this increased site‐specific methylation may in turn impact socioemotional function.

### 2.6 Surgery

In our cohort studies, we found that surgery early in life significantly contributed to poorer brain maturation in the neonatal period^34^ and at school age,^48^ over and above pain in the NICU and other clinical factors related to prematurity. In landmark studies, Suellen Walker and colleagues found that differences in tactile and pain thresholds at age 11 years between children born extremely preterm compared with full‐term were explained by early surgery persisting to age 19 years.^53^ Long‐term effects of early surgery may be centrally mediated. Unraveling to what extent prior clinical factors, pain management during and post‐surgery, pharmaceutical agents, other events during surgery such as periods of reduced brain oxygenation contribute to these long‐term changes remains a challenge to be addressed.

### 2.7 Pharmacologic pain management

Morphine is widely used in NICU care to treat ongoing pain/stress and distress of mechanical ventilation. There are long‐standing concerns that exposure to opiates may have adverse effects on the immature brain. On the whole, studies have been re‐assuring that morphine treatment is not related to severe brain injury or major impairments (see McPherson & Grunau^54^ for a review). However, in our cohort studies, greater internalizing (anxiety/depressive) symptoms have been associated with higher morphine exposure.^50^, ^55^ In contrast to early optimism by others that pre‐emptive pain treatment with morphine might prevent adverse effects of NICU pain, we have found that morphine does not protect the immature developing brain,^34^, ^43^, ^56^ HPA axis,^49^ or child self‐ratings of pain sensitivity^16^ from effects of neonatal pain/stress. Recently, we examined a panel of genes related to morphine metabolism, and found promising markers of risk for internalizing behavior.^50^

### 2.8 Parenting stress and interaction

We and others have shown that infants born very preterm are more affected by the parenting context than full‐terms, across infancy and early childhood. Parenting stress is high in these parents^29^ persisting long after NICU discharge, and is in part a reflection of realistic concerns about their child's development in the face of poorer progress.^57^ Importantly, our work found parent sensitivity to child cues at least partially modulates adverse effects of neonatal pain.^29^, ^31^, ^58^ This is consistent with work from other groups showing that higher‐quality parent‐infant interaction mediates the relationship between perinatal risk and neurodevelopment.^59^ There has been a revolution in NICU care to encourage parent involvement, skin‐to‐skin contact, music therapy, mother‐infant smell. An integrated approach training and supporting parents in the NICU how to recognize and reduce their infant's stress and pain, including components of parent contact such as skin‐to‐skin care, shows early promise through more mature brain connectivity.^60^, ^61^

## 3 CONCLUSION

It is an honor to contribute personal perspectives from my research career. My reflections have emphasized positive opportunities across the years. But, to encourage young clinician‐scientists, I must mention that sometimes the way forward was not easy, and took twists and turns. Patience and respectful persistence, with an eye on long‐term goals, are keys to establishing new research in complex medical settings. Approaching and listening to the bedside clinicians are really important for solving apparent roadblocks. Finding the right collaborators can be challenging.

Research in pediatric pain has been highly rewarding, especially seeing the major changes in policy and practice due to studies that established pain perception early in life, and potential sequelae. It has been a privilege to contribute to understanding that repetitive exposure to procedural pain in the NICU can have long‐term and widespread impacts. Alterations in brain development, stress regulation, and epigenetic processes are some of the biologically plausible potential pathways implicated. There is now converging evidence that the developing very preterm brain is exquisitely sensitive to pain/stress during a “critical window” in early life. Moving forward, it is essential to consider possible sex‐specific mechanisms underlying long‐term effects of pain in very preterm infants. The next critical frontier in the field of pain in preterm infants is to evaluate which pain treatments protect the developing brain.

## CONFLICTS OF INTEREST

The author declares no conflicts of interest.

## AUTHOR CONTRIBUTIONS

RE Grunau wrote this manuscript.
